# Antibody Conjugates-Recent Advances and Future Innovations

**DOI:** 10.3390/antib9010002

**Published:** 2020-01-08

**Authors:** Donmienne Leung, Jacqueline M. Wurst, Tao Liu, Ruben M. Martinez, Amita Datta-Mannan, Yiqing Feng

**Affiliations:** 1 Biotechnology Discovery Research, Lilly Research Laboratories, Lilly Biotechnology Center, Eli Lilly and Company, San Diego, CA 92121, USA; 2 Discovery Chemistry and Research Technology, Lilly Research Laboratories, Lilly Biotechnology Center, Eli Lilly and Company, San Diego, CA 92121, USA; jwurst@lilly.com (J.M.W.); liu_tao2@lilly.com (T.L.); Martinez_ruben_martin@lilly.com (R.M.M.); 3 Exploratory Medicine & Pharmacology, Lilly Research Laboratories, Lilly Corporate Center, Eli Lilly and Company, Indianapolis, IN 46225, USA; datta_amita@lilly.com; 4 Biotechnology Discovery Research, Lilly Research Laboratories, Lilly Technology Center North, Eli Lilly and Company, Indianapolis, IN 46221, USA; feng_yiqing@lilly.com

**Keywords:** antibodies, site-specific conjugation, bioconjugates, ADC, antibody-drug conjugates, payloads, linkers, nucleic acids, ADME, developability, formulation

## Abstract

Monoclonal antibodies have evolved from research tools to powerful therapeutics in the past 30 years. Clinical success rates of antibodies have exceeded expectations, resulting in heavy investment in biologics discovery and development in addition to traditional small molecules across the industry. However, protein therapeutics cannot drug targets intracellularly and are limited to soluble and cell-surface antigens. Tremendous strides have been made in antibody discovery, protein engineering, formulation, and delivery devices. These advances continue to push the boundaries of biologics to enable antibody conjugates to take advantage of the target specificity and long half-life from an antibody, while delivering highly potent small molecule drugs. While the “magic bullet” concept produced the first wave of antibody conjugates, these entities were met with limited clinical success. This review summarizes the advances and challenges in the field to date with emphasis on antibody conjugation, linker-payload chemistry, novel payload classes, absorption, distribution, metabolism, and excretion (ADME), and product developability. We discuss lessons learned in the development of oncology antibody conjugates and look towards future innovations enabling other therapeutic indications.

## 1. Introduction

**Figure 1:**
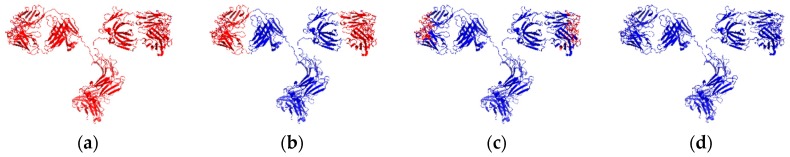
The evolution of (**a**) murine, (**b**) chimeric, (**c**) humanized, and (**d**) fully human monoclonal antibodies through protein engineering. Red and blue represents mouse and human antibody sequence respectively. The antigen binding complementarity determining regions (CDRs) are shown as sticks. The new generation of antibody-drug-conjugates (ADCs) utilized humanized (**c**) and fully human antibodies (**d**).

Since the first monoclonal antibody drug approval (OKT3) in 1986, over 60 antibody therapeutics have become marketed drugs to date [1]. The number of protein therapeutics entering clinical development, including antibodies, antibody fragments, bispecifics, Fc-fusion proteins, and antibody-drug conjugates is expected to grow due to robust pipelines and high success rates for treating various diseases [2,3]. With the advances and extensive experience in antibody engineering over the past decades [4], antibody therapeutics have evolved from murine (e.g., OKT3) to chimeric (e.g., Rituxan^®^) to fully human (e.g., Humira^®^) as depicted in Figure 1. Monoclonal antibody-based therapeutics have been built to deliver specific effector functions or as bispecifics and conjugates to achieve the desired pharmacological effects [5,6]. Antibody discovery was enabled by murine hybridoma technology [7] followed by humanization [8] to deliver therapeutic antibodies with lower risk of immunogenicity [9]. Display technologies and transgenic animals have pushed the boundaries to produce antibodies with fully human sequences [10]. Antibody conjugates have similarly taken advantage of the progress made in monoclonal antibody development and improvements in conjugation chemistries [11,12,13] to expand the druggable target space for antibody-based therapies. These advances in antibody development are crucial to the success of antibody conjugates.

While chemotherapy and radiation have been the dominant treatments of cancer for decades, their lack of ability to distinguish between healthy and tumor cells has fueled the desire to create tumor specific delivery of cytotoxic payloads and radionuclides via antibody conjugates. Oncology antibody conjugates have successfully delivered potent chemotherapeutic and radioactive agents to kill tumor cells [12]. Currently, all of the FDA approved antibody-drug-conjugates (ADCs) are targeted cancer therapies (Table 1) [14], including the latest approval in June 2019 for Polivy^®^ [15]. Herein, we review the progress made in oncology ADCs in terms of conjugate design and development, linker payload conjugation chemistries and highlight novel non-oncology conjugate innovations.

## 2. Critical Considerations for Antibody Conjugates

First generation oncology ADCs in the 1990s were based on murine or chimeric antibodies which were plagued with immunogenicity issues [16] and linker instability [17]. Immunogenicity of protein therapeutics has a critical impact on the pharmacokinetics and drug disposition and ultimately clinical success [18,19]. These molecules were designed to deliver a variety of protein toxins [20] and microtubule binding drugs [21] as the cytotoxic payloads. Limited antigen density on tumors, low potency of the payloads, and the low average drug-antibody-ratio (DAR ~3–4) prevented efficacious quantity of drug delivered, which was proposed to be one of the reasons for initial ADC failures, while higher DAR conjugates suffered from toxicity and low therapeutic index. Second generation ADCs from the last 10+ years approved by the FDA were armed humanized antibodies coupled to stabilized linkers and more potent payloads, such as auristatins, calicheamicins, and maytansinoids (Table 1).

In an ideal situation, ADC payloads should be inactive in circulation when conjugated to an antibody via a linker and remain stably conjugated until the conjugate reaches the target of interest. Upon internalization of the conjugate-target complex, active payload is released inside target cells after lysosomal degradation of the linker or the antibody itself. In addition to reducing target-independent uptake, conjugate stability remains crucial for specific delivery and distribution of payload to the target tissue from systemic circulation. Conjugation sites, chemistries and linker designs coupled with DAR load greatly affect plasma stability, biophysical properties, and consequently pharmacokinetics of the conjugate. Next generation ADCs will likely incorporate fully human antibodies with site-specific conjugation and novel linkers to reduce immunogenicity and optimize biodistribution and payload delivery. These topics will be discussed in this review.

### 2.1. Target and Antibody Selection

One of the key contributing factors to clinical failures has been the bio-distribution of an ADC, which is critically dependent on the relative target expression as well as target-independent uptake. Other aspects such as conjugate and linker stability and payload properties are described in other sections. Preferably for an oncology treatment with a biologic, antigen targets should have high expression levels on tumor cells and little to no expression on normal tissues. Internalization of the target-antibody complex is crucial for specific intracellular release of payloads. Antibodies are ideal delivery vehicles due to their high specificity to targets and long half-life, which is the result of pinocytosis and subsequent neonatal Fc receptor (FcRn)-mediated recycling [22]. Prolonged systemic circulation enables conjugate accumulation at the target sites.

The antibody Fc choice is an important consideration for both monoclonal antibody therapeutics and ADC therapeutics [23]. Fc-mediated effector functions such as antibody-dependent cellular cytotoxicity (ADCC) or complement-dependent cytotoxicity (CDC) are part of the mechanism of action for depleting antibodies [24]. However, with ADCs, the contribution of effector functions to efficacy and toxicity are not well understood. It is noted that two out of the five currently FDA approved ADCs (Mylotarg^®^ and Besponsa^®^) employed IgG4 antibodies which lack effector functions. Although the effector functions have the potential to augment the anti-tumor activities of the ADCs, engaging Fcγ receptors is also a possible cause for off-target and dose-limiting toxicity (reviewed in [25].) Emerging literature suggests that the antibody internalization and delivery of the toxic drug to the target cells serves as the primary mechanism of action for ADCs that is far more efficient than ADCC and Antibody-Dependent Cellular Phagocytosis (ADCP). For example, trastuzumab-DM1, SYD985, and DS-8201a all target HER2 and have shown similar ADCC activity as trastuzumab but they have demonstrated dramatically more anti-tumor activity than trastuzumab [26,27,28,29,30,31]. The anti-Trop-2 ADC IMMU-132 represents a more striking case as this ADC lost 60%–70% of the ADCC activity compared with the unconjugated mAb upon the conjugation of SN38 [32]. Nevertheless, this ADC demonstrated significant antitumor effects in mice bearing human pancreatic or gastric cancer xenografts [32] and is showing promise in clinical trials [33,34]. On the other hand, it is well established that afucosylated IgG1 increased binding to FcγRIIIa on effector cell such as natural killer cells and led to enhanced ADCC activity [35]. An example is GSK2857916, an afucosylated IgG1 antibody as a non-cleavable MMAF conjugate targeting B-cell maturation antigen (BCMA), in the clinic for multiple myeloma and demonstrated potent anti-tumor activity while it harnessed multiple cytotoxic mechanisms [36,37].

Due to the large size of an antibody conjugate, the stromal barrier [38] and tumor tissue penetration is an obvious obstacle for oncology ADCs to overcome for the treatment of solid tumors. Nonetheless, the successful targeting and delivery of payload to liquid tumor in circulation pushed the concept and led to a new frontier in ADCs to deliver small molecules for non-oncology indications [39,40]. Novel non-cytotoxic payloads have been conjugated to antibodies in the hopes of extending the pharmacokinetic properties and increasing therapeutic index of the drugs. Genentech has pioneered antibody-antibiotic conjugates [41,42] to target intracellular *Staphylococcus aureus* within host cells. Others have leveraged the internalization mechanism of antibodies to deliver immunosuppressive, cardiovascular or metabolic disorder small molecule drugs to specific cells using cell surface targets such as E-selectin [43], CD11a [44,45], CD25 [46], a3(IV)NC1 [47], CXCR4 [40,48], CD45 [49], CD70 [50], CD74 [51], and CD163 [52,53]. Examples of linker payloads as well as formulation and delivery challenges for non-oncology indications are discussed below. Additionally, genes of interest have been targeted in specific cell types to produce durable response using antibody-oligonucleotide conjugates [54,55]. Delivery of oligonucleotides have traditionally been challenging and various modifications have been employed to facilitate better cell penetration. This is explored in a later section.

### 2.2. Conjugation Methods

**Figure 2:**
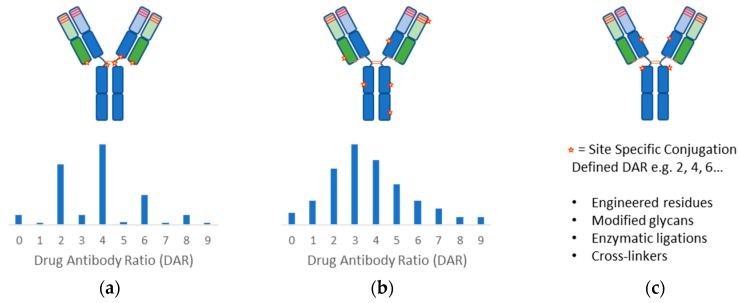
Antibody conjugation methods include (**a**) cysteine-reactive, and (**b**) lysine-reactive chemistries which generate heterogeneous mixtures of drug-antibody-ratio (DAR), while (**c**) site specific conjugation methods deliver more homogeneous product with defined DAR using engineered residues, modified glycans, enzymatic ligations, and chemical cross-linkers. Schematic representation of antibody heavy chains and light chains are colored blue and green respectively. complementarity determining regions (CDRs) and conjugation sites are depicted as red bars and stars respectively. Approximate DAR distribution for stochastic cysteine and lysine conjugations are presented as bar charts.

Antibody conjugation methods (Figure 2) have been extensively reviewed [11,56,57,58]. To date, all the FDA approved ADCs have relied on coupling reactions using either the nucleophilic primary amino group of surface-exposed lysines or the thiol group of reduced structural disulfides. The resulting product is a controlled heterogeneous mixture of antibodies with average drug load. High DAR species leads to aggregate formation, lower tolerated dose, and faster systemic clearance while low DAR species suffer from low efficacy [59]. Although DAR profile can be controlled by conjugation process development and specific DAR can be purified, site-specific methods to produce more homogeneous drug products would improve yield and biophysical properties, which will be critical for the next generation of ADCs. Towards these ends, extensive experience in protein engineering has allowed strategic placements of residues at specific locations enabling chemo-selective conjugation reactions. Researchers at Genentech first demonstrated that conjugation stability is location dependent and specific engineered cysteine sites were able to improve therapeutic index [60,61,62]. Cysteine insertions at specific sites can also efficiently produce stable conjugations [63]. Others have shown similarly that location of the conjugation sites can impact the stability and pharmacokinetics of the ADCs using alternative residues and chemistries [64,65].

Enzymatic methods have also been explored (reviewed in [66]) where recognition sequences have been engineered into the antibody to facilitate site-specific conjugation. Most well-exemplified in this category are enzymes such as transglutaminase [65,67,68,69], sortase [70,71,72] and formylglycine-generating enzyme (FGE) [73,74]. Transglutaminases (TG) catalyze a stable isopeptide bond between an amine of a lysine and the γ-carbonyl amide of a glutamine. Deglycosylation of N-linked glycan on a native antibody exposes glutamine at position 295 for site-specific conjugation with TG either through direct coupling with an amine-functionalized linker payload or via a two-step coupling by installing bio-orthogonal azide or thiol for strain-promoted azide-alkyne cycloaddition and maleimide chemistry respectively [67]. Alternatively, glutamine residues can be engineered and short glutamine (LLQG) tags were introduced into different regions to yield highly stable site-specific conjugates with good pharmacokinetic profiles [65,68,69]. Sortase catalyzes a transpeptidation reaction between a N-terminal glycine of GGG peptide or linker payload with the threonine-glycine bond in a LPXTG motif to produce a peptide fusion or site-specific ADC with high in vitro and in vivo potency [70,71,72]. Lastly, SMARTag^®^ [75] is an example where formylglycine-generating enzyme (FGE) converts an engineered cysteine residue in a specific peptide sequence to produce an aldehyde tag in cell culture [73,74] to enable conjugation with linkers via oxime formation or a Pictet–Spengler reaction [76,77].

Other conjugation chemistries involved the engineering of unnatural amino acids [78,79,80,81,82] to install reactive groups in the antibody for bio-orthogonal chemistry [77]. Companies such as Ambrx [81] and Sutro Biopharma [83] have utilized these elegant approaches to generate site-specific ADCs. An orthogonal amber suppressor tRNA/aminoacyl-tRNA synthetase pair is used to incorporate the unnatural amino acids such as para-acetylphenylalanine (pAF), para-azidophenylalanine (pAZ), and para-azidomethylphenylalanine (pAMF) into recombinantly expressed antibodies in cell-based or cell-free systems. Reactive ketone in pAF forms a stable oxime linkage with alkoxyamine containing linkers, while the azido group in pAZ and pAMF undergoes click chemistry with alkynes to produce homogenous ADCs.

Alternatively, native antibodies can be conjugated site-specifically [84] after enzymatic modification of natural amino acids such as tyrosines [85] and glutamines [67], and carbohydrates can be oxidized chemically or modified with enzymes to produce reactive groups for conjugation [86,87,88]. For instance, sodium periodate oxidation of fucose at the native N-linked glycan of an antibody installed an aldehyde for conjugation with hydrazides [86]. Glyco-remodeling methods include enzymatic transfer of galactose and sialic acid with a mixture of transferases yielded glycans which can be oxidized with periodate to form oxime conjugates with aminooxy linker payloads [87], while sialytransferase can also incorporate an azide modified sialic acid derivative into the antibody for click chemistry [89]. Similarly, SynAffix BV utilized a 2-steps GlycoConnect™ process to trim a mixture of glycoforms with endoglycosidase, followed by enzymatic transfer of azido sialic acid for copper-free click chemistry [88]. Lastly, site-specific conjugation approach to retain the structural stability of a native antibody is to cross-link the reduced interchain disulfides with re-bridging chemical reagents [90,91,92,93,94].

Taken together, the various novel site-specific conjugation methods often require additional investments in manufacturing processes to produce the clinically viable products at large scale. Many development advances have been made in site-specific conjugations enabled a new generation of ADCs to enter the clinic in recent years, but this topic is outside the scope of this review.

## 3. Current Small Molecule Payloads and Beyond

**Figure 3:**
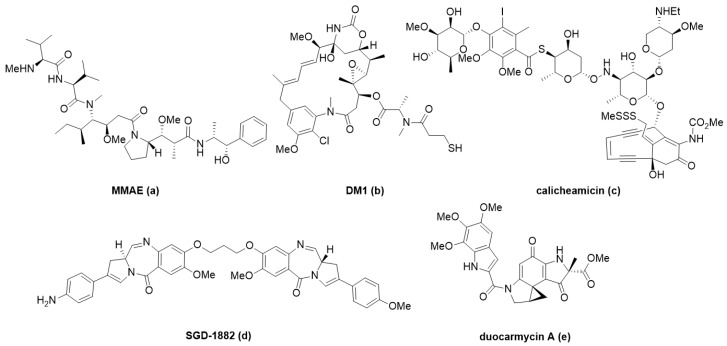
Examples of ADC payloads used clinically include, monomethyl auristatin E (MMAE, **a**, emtansine (DM1, **b**), calicheamicin (**c**), pyrrolobenzodiazepine dimer (PBD, SGD-1882, **d**), and duocarmycin A (**e**).

ADC payloads have been mostly anti-mitotic small molecules for oncology indications [13,39]. The clear advantage of conjugates in this space is targeted delivery at efficacious doses that are below what could be given systemically due to the toxicity of the payload. Current approved ADCs such as Adcetris^®^, Kadcyla^®^, and Polivy^®^ carry cytotoxic payloads that rely on the anti-mitotic mechanism of action (MOA) such as monomethyl auristatin E and emtansine (Table 1; Figure 3a,b, respectively). This class of payloads still predominates in clinical stage research, including novel derivates of these small molecules, despite efforts to use payloads with alternative MOAs [13,39,95].

**Figure 4:**
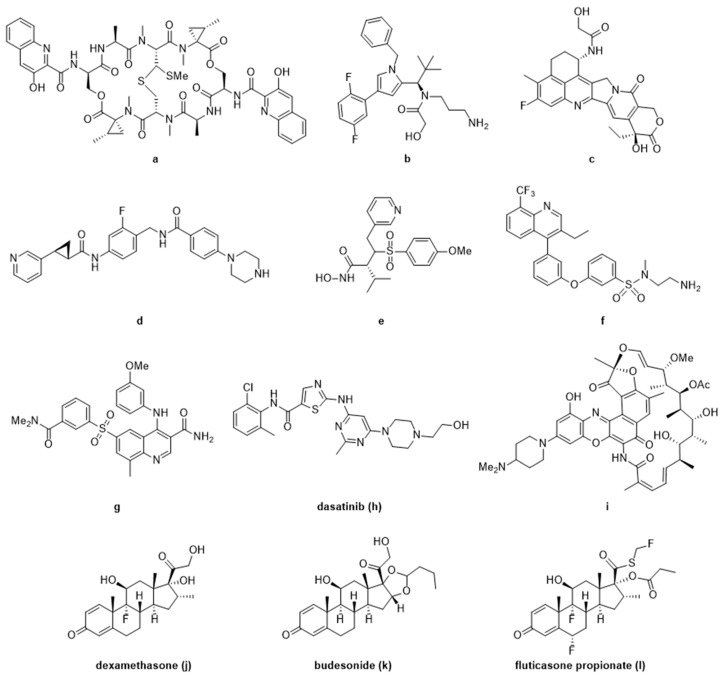
Expanding payload space in oncology with DNA disrupting bis-intercalator depsipeptide (SW-163D, **a**), pyrrole-based kinesin spindle protein (KSP) inhibitor (**b**), topoisomerase I inhibitor (DXd, **c**), nicotinamide phosphoribosyltransferase (NAMPT) inhibitor (**d**), and MMP9 inhibitor (CGS27023A, **e**). Examples of non-oncology payloads include LXR agonist (**f**), PDE4 inhibitor (GSK256066, **g**), kinase inhibitor dasatinib (**h**), antimicrobial rifamycin analog (**i**), GR agonists dexamethasone (**j**), budesonide (**k**), and fluticasone propionate (**l**).

DNA damaging agents are another class of well-studied payloads [96,97,98]. Enediynes, such as calicheamicin **c**, are the warhead in Mylotarg^®^ and Besponsa^®^ (Table 1, Figure 3). They act through DNA-binding and induction of DNA-double strand breaking to produce a cytotoxic response in target cells. Other important DNA damaging agents currently used as payloads in clinical trials are duocarmycins **e** and pyrrolobenzodiazepines (PBD, **d**); they have unique, well-understood minor groove binding mechanisms that disrupt normal DNA function leading to cell death. Novel payloads have been explored recently in this space such as bis-intercalator depsipeptides with nanomolar affinity to DNA [99]. Pfizer demonstrated that this ultra-potent payload **a** (Figure 4) can overcome the previous limits of efficacy in animal models, therefore, expanding their relevance to indications beyond liquid tumors.

Further, highly-potent payloads described in the conjugate space (Figure 4) are pyrrole-based KSP (kinesin spindle protein) inhibitors **b** [100], which are traditional in the sense of their antimitotic mechanism-of-action, but newly incorporated pyrrole functionality provided an increase in efficacy against a wide-range of cancers previously untouched by this class. Continuing the push beyond the limits of first-generation payloads, Daiichi Sankyo^®^ has incorporated a topoisomerase I inhibitor, exatecan derivative (DXd, **c**) into an ADC (DS-8201a). With superior pharmacodynamic and safety properties, in large part from the payload, DS-8201a has shown promising response in trastuzumab emtansine-insensitive cancers [31].

As with other oncology examples, nicotinamide phosphoribosyltransferase (NAMPT) inhibitors **d** have not succeeded in the clinic due to low therapeutic index with limiting toxicities, but exploitation of ADC targeted delivery of NAMPT inhibitors provides an outlet for these potent payloads [101] in preclinical studies. Similarly, an anti-MMP9 antibody was conjugated to a non-selective MMP inhibitor (CGS27023A, **e**), showing remarkable selectivity for MMP9 alone, due to the antibody targeting in vitro [102].

Beyond oncology, ADCs are just beginning to show promise as a novel modality, offering a potential solution to the pitfalls of traditional drug discovery. Using an anti-CD11a antibody conjugated to an LXR (Liver X Receptor) agonist **f**, researchers were able to target macrophages to reverse cholesterol transport and reduce inflammation without negatively affecting hepatocytes, which have previously shown on-target toxicity with LXR agonists [44]. Antibody targeting CD11a was also used to selectively deliver a known PDE4 (Phosphodiesterase 4) inhibitor (GSK256066, **g**) and reduce inflammatory cytokine production, showing promise for the treatment of chronic inflammatory conditions with a more optimal therapeutic index [45]. In a similar fashion, dasatinib **h**, a known Src-family kinase inhibitor against leukemia was re-purposed as an immunosuppressive ADC using an anti-CXCR4 antibody to specifically target T-cells without undesirable side-effects [48].

Antimicrobial research is another area demanding novel approaches; it is well established that our current arsenal against microbes is failing and the discovery of new molecules has been limited [103]. Genentech pioneered the antibody-antibiotic conjugate (AAC) to deliver a rifamycin analog **i** intracellularly via an anti-*S. aureus* antibody which demonstrated marked clearance of latent bacteria reservoirs thought to be the cause of recurring infection [41].

Other known classes of molecules, namely, glucocorticoids are used as a standard of care for many immunological indications. They come with less-than-desirable side-effects at efficacious doses [104,105] with chronic use. One of the first examples of targeted glucocorticoid delivery via antibody used an anti-E-selectin conjugate to deliver dexamethasone to TNFα stimulated endothelial cells [43]. These early proof-of-concept experiments tracked conjugate internalization, intracellular release of steroid, and reduction of the pro-inflammatory IL-8 expression. Expanding on these preliminary experiments, an anti-CD163 conjugate was used to deliver dexamethasone to macrophages showing a synergistic anti-inflammatory effect of the conjugate versus its components alone, and a significant reduction in the systemic steroidal side-effects of orally dosed dexamethasone at the same efficacy [52]. Known glucocorticoid receptor (GR) agonists (i.e., dexamethasone **j**, budesonide **k**, and fluticasone propionate **l**) have also been attached to anti-CD74, anti-CD70, and anti-CD25 antibodies showing an immune cell targeted anti-inflammatory response, as well as highlighting the complexities of developing ADCs in this therapeutic area [46,50,51,106]. This novel modality promises treatment to larger populations of patients with autoimmune disorders, in a disease specific fashion that could potentially replace traditional steroid treatments as the standard of care.

## 4. Nucleic Acid Conjugates

For traditional oncolytic ADCs, the challenge of delivery manifests in the form of systemic toxicity of the potent cytotoxic payloads. However, similar molecular calculus may be applied to extensively cleared molecular entities which may otherwise have trouble reaching the tissues of interest. A rapidly-advancing molecular space which is typically impeded by delivery issues is that of synthetic therapeutic oligonucleotides including antisense oligonucleotides (ASOs) and short interfering RNA (siRNA) [107,108,109].

While traditional ADC payloads are small molecules which act on cellular machinery to elicit their desired phenotype via action on a molecular target, the current generation of synthetic oligonucleotide therapeutics instead act on information molecules upstream of their targets such as endogenous mRNA, achieving specificity through base-pair complementarity. Several different mechanisms of action have been clinically validated. Examples include intronic splice modulation (i.e., nusinersen, an ASO for treating spinal muscular atrophy) and formation of a catalytic mRNA silencing complex (i.e., patisiran, a lipid-nanoparticle formulated siRNA for treating ATTR amyloidosis). The details of these mechanisms, along with others, have been recently reviewed [110] and are beyond the scope of this discussion. The chemical structures and properties of oligonucleotides that make this therapeutic space challenging, however, are central to this discussion; their poor tissue penetration and circulation half-life are often attributed to the polyanionic backbone characteristics [111,112].

**Figure 5:**
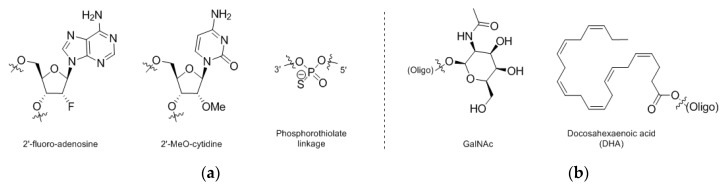
(**a**) stabilizing chemical modifications for siRNA, (**b**) oligo delivery conjugate moieties.

A number of advances in both oligonucleotide chemistry as well as conjugate chemistry have been enabling oligonucleotide clinical candidates as a class and will likely impact oligonucleotide bioconjugates [113]. For example, it has been demonstrated that appending of N-acetylgalactosamine (GalNAc, Figure 5b) residues to the end of siRNA strands allows for efficacious loading of hepatocytes in vivo with sustained target knockdown [114]. Along with GalNAc, number of other common modifications in oligonucleotide chemistry have been utilized, such as 2′-modification (i.e., 2′-F and 2′-OMe nucleosides, Figure 5a) and sulfurized phosphate analogs (i.e., phosphorothiolates, Figure 5a). Similar themes of heavy chemical modification have been utilized in ASOs. For example, one report principally noted that GalNAc conjugation ameliorated some nephrotoxicity signals in PCSK9-targeting ASOs, with data suggesting such conjugation could be a path towards addressing oligo-induced nephrotoxicity concerns more generally [115]. Beyond GalNAc, other oligonucleotide conjugation strategies have been found effective pre-clinically in targeting specific cellular populations or promoting systemic availability, as with conjugation of a GLP1R agonist [116] or conjugation to various lipids such as cholesterol or docosahexaenoic acid (DHA, Figure 5b) [117], respectively.

Although there are limited examples of discreet therapeutically-oriented antibody-oligonucleotide conjugates in the literature, the current studies have made significant headway and put to use many of the strategies discussed herein. In an early example reported, hu3S193, an internalizing humanized Lewis-Y mAb was conjugated to a largely unmodified STAT3 siRNA [118]. This conjugation by utilizing non-specific amino residue labeling of hu3S193 with activated hydrazonal nicotinamide (HyNic) reagent which was covalently coupled with STAT3 siRNA using an aldehyde linker. Cellular specificity was confirmed through flow cytometry and internalization by confocal microscopy, the construct could only effect STAT3 knockdown when high doses (100 µM) of chloroquine as an endosomal disrupting agent was added.

The disconnect between specific internalization and siRNA target knockdown was further demonstrated in a systematic study which applied a number of important antibody-drug conjugate parameters [119], which included variations of linker chemistry, cell surface receptor identity, receptor internalization types and count, antibody linkage positions, and antibody formats. This study utilized several sophisticated methods, including the application of site-specific engineered cysteine conjugation (Genentech’s THIOMAB™ platform) to deliver reproducible and largely homogeneous conjugates, as well as highly modified chemically-stabilized housekeeping gene (PPIB) siRNA constructs to enable in vivo studies. While seven different internalizing antigens were profiled, only three showed any knockdown of the target gene and of those only TENB2 on cell lines with high surface receptor density was able to achieve knockdown levels of greater than 50% in vitro. It was inconclusive what properties of the different active conjugates enabled effective knockdown, but importantly, conjugates were able to demonstrate 33% transcript knockdown with cellular specificity to tumor cells near the vasculature in a mouse xenograft model.

At this time, there have yet to be any clinical studies on antibody-oligonucleotide conjugates, but several of the requisite preclinical proof-of-concept studies have been reported. One notable example has demonstrated application of a myostatin-silencing siRNA-antibody conjugate in a mouse model of muscular regeneration [120]. In this study, the well-profiled CD71 receptor (transferrin receptor) was chosen for muscular target engagement utilizing anti-CD71 Fab’-siRNA conjugates. In profiling methods of administration, principal findings were that equivalent target engagement was achievable through different perfused systemic administration routes. PCR-based detection methods were used to verify conjugate was detectable 24 h post-dose, but notably durable silencing up to a month out was observed. Additionally, intramuscular injection enabled superior levels of target engagement in a model of peripheral artery disease, with muscular regeneration due to myostatin knockdown observed with microgram-scale injections. While this Fab’-based study utilized structural cystines for conjugation, other therapeutic antibody-oligonucleotide conjugation systems not yet described in this section have been reported, including two oncology examples which applied two-step conjugations for azide-labeling the antibody which were then treated with cyclooctyne-appended ASOs to generate the conjugates [121,122]. Other antibody-oligonucleotide conjugation methods beyond the scope of this review (i.e., for immuno PCR applications, pre-targeted radiotherapy, or those which utilize non-covalent heterogeneous complexes) have been recently reviewed [109].

While these results are promising, further analytical work must demonstrate pharmacokinetic profiling of these conjugates to enable clinical study. In this vein, a report describing a triplex forming oligonucleotide ELISA assay utilized locked nucleic acid-containing probes [123] for conjugate quantification. This assay is distinguished from PCR-based methods as a direct, quantitative readout of intact oligonucleotide-antibody conjugate. The authors demonstrated that this assay is capable of accurately detecting conjugate doped into cellular matrices such as serum or tissue homogenate down to a limit of detection of 120 pg/mL. These early developments in antibody-oligonucleotide conjugates can help propel the next wave of novel conjugates to leverage the cellular specificity of antibodies and target-gene specificity of oligonucleotide therapies.

## 5. Linkers

While a simple concept at first glance, a linker is far more complex than a mundane spanning element between the small molecule payload and the antibody which make up the ADC. It ensures the fundamental principles of targeted drug delivery of ADCs-minimizing premature drug release in plasma and promoting selective release of payload to the target cell. Additionally, it can modulate the physiochemical property of the overall conjugate. This requires the linker design to be stable in circulation and upon antibody-mediated internalization, the payload is efficiently released.

**Figure 6:**
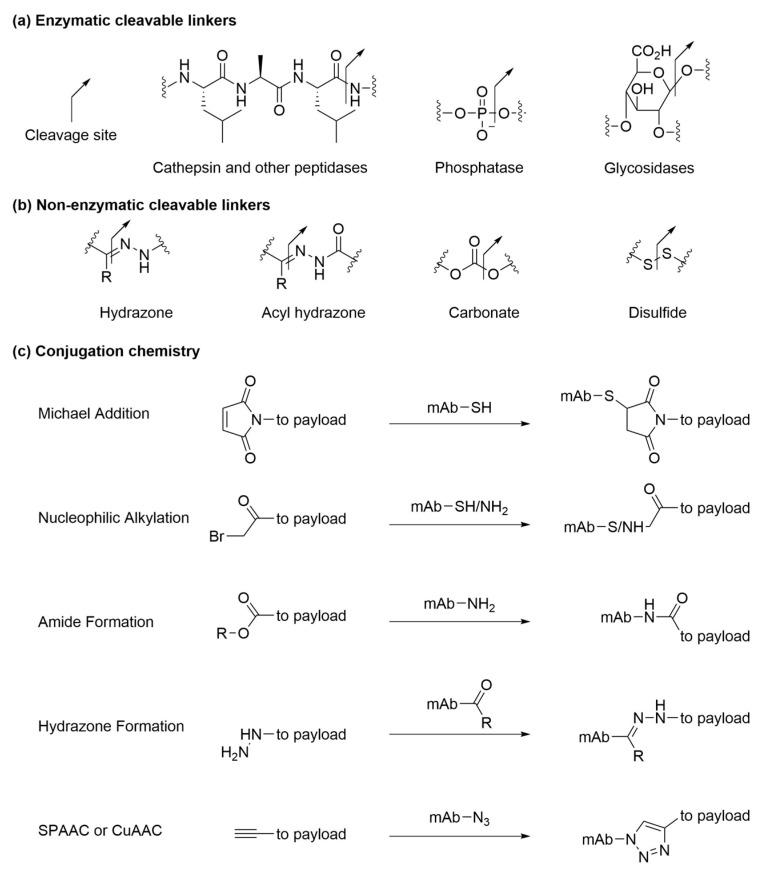
(**a**) Enzymatic cleavable linkers; (**b**) non-enzymatic cleavable linkers; (**c**) conjugation chemistry.

To meet the desired therapeutic effect, cytotoxic payloads can be designed to be released either intracellularly or extracellularly [10,13,124,125,126]. For instance, intracellular hydrolytic enzymes can recognize specific linker motifs (Figure 6a) and catalyze the cleavage reactions to release payloads inside endosomes or lysosomes. Cathepsin family enzymes and other proteolytic enzymes are responsible for the digestion of peptide linkers in lysosomes [69]. Selection of the linker peptide sequence affects the plasma stability of ADCs and the efficiency of proteolytic cleavage of linkers in target cells [127,128]. Phosphatases [106] and glycosidases [129,130] are other examples of hydrolytic enzymes that are present in lysosomes at high concentrations and break down respective linkers of internalized ADCs. Alternatively, through exploitation of the relatively acidic and reductive tumor microenvironment, payloads may also be released extracellularly through non-enzymatic cleavable linkers (Figure 6b) at a tumor site. For example, hydrazine and acyl hydrazine (approved products in Table 1) [25,131], ketal and acetal [132], as well as carbonate [133] are acid-labile linkages that are designed to degrade at low pH in cell chambers (pH 5.5 in endosome, pH 4.5 in lysosome) and remain intact in circulation (pH 7.4). Disulfide linkages are subjected to glutathione attack in cytosol, thus offering a chance of selective releasing toxic payloads in tumor cell. How the conjugation site and steric hindrance around disulfide linkage modulates its stability in plasma is a subject that has attracted extensive studies [134,135].

Both enzymatic cleavable linkers and non-enzymatic cleavable linkers have been conjugated onto antibodies through naturally occurring cysteine and lysine residues (Figure 6c). Nucleophilic thiol groups can react with maleimide and alkyl-halide to produce stable conjugates. In cases of maleimide-based ADCs where stability is a concern, semi-hydrolysis of maleimide has been reported as an effective strategy to minimize the retro-Michael reaction of thiomaleimide and preventing premature payload loss [136]. Amino groups on surface exposed lysines can form stable amide via alkylation of an activated ester on the linker. Side chains of natural amino acids can be modified or engineered to produce ketones to form imines or hydrazones. Notably, recent site-specific ADCs utilize novel linker conjugation chemistries with unnatural amino acids that are incorporated on engineered antibodies through imine/hydrazine formation, copper-catalyzed azide alkyne cycloaddition (CuAAC), and strain promoted azide alkyne cycloaddition (SPAAC) [44,78,80].

**Figure 7:**
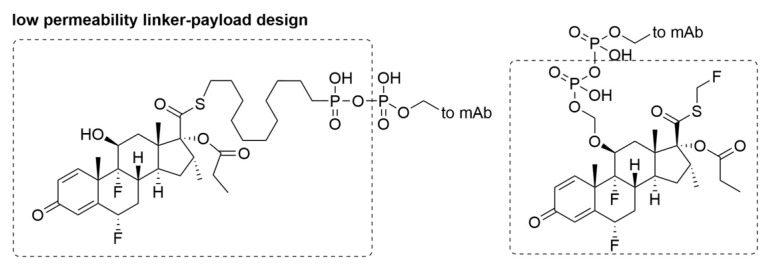
Low permeability linker-payload design.

In sharp contrast to approved cytotoxic ADCs for oncological indications, the goal of ADCs for non-oncological applications is the selective modulation of target cells without on-target adverse bystander effects. Besides selection of non-toxin-based payloads, this new direction also demands new concepts in linker-payload design. Linker-payload design is critical in modulating bystander effect of payloads. A diphosphatase-cleavable linker has been reported for the selective delivery of immune suppressing payloads to immune cells following ADC internalization and diphosphate cleavage (Figure 7) [51]. In this study, the fluticasone propionate derived payload has a high intrinsic binding affinity to target, but also bears a charged phosphate moiety. Due to antibody-driven delivery and limited free payload permeability, the target exposure of payload to cell is increased, and a superior in vitro potency is observed. Further in vivo testing may require additional linker development.

**Figure 8:**
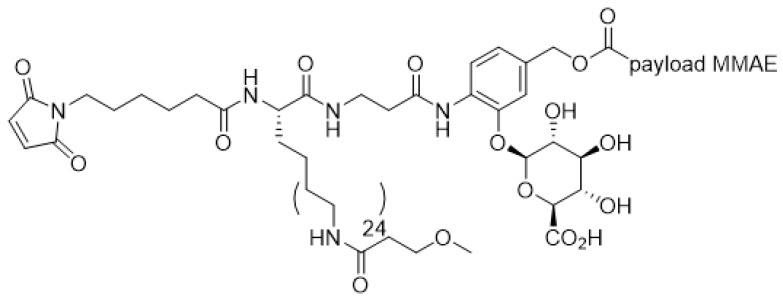
Hydrophilic linker-payload design.

Linker-payload design is also critical to the successful implementation of high-DAR ADCs, via reduced aggregation and improved overall pharmacokinetics profiles. Even though increasing DAR instinctively increases in vitro potency of ADCs, it may not translate to an improvement of in vivo potency since the plasma clearance of ADCs rises along with DAR [129]. The aggregation and fast clearance problem may be largely mitigated without extensive linker optimization for water-soluble payloads such as the topoisomerase I inhibitor DXd in DS8201a, which is an ADC with DAR 8 that has shown remarkable in vivo stability both pre-clinically and clinically [31]. For highly hydrophobic payloads, the paradoxical effect of higher DAR resulting in lower exposure can be corrected by novel linker design. Several linker modifications to enhance hydrophilicity have been reported that allowed ADC to be produced with DAR as high as 8 (reviewed in [124]). These modifications include addition of a polyethylene glycol (PEG) moiety [127,128], a glucuronic acid unit [129], or a combination of branched PEG moiety and glucuronic acid unit [130]. For instance, hydrophilic linker construct in Figure 8 [129] minimizes the detrimental hydrophobicity associated with increasing DAR, imparting an optimal pharmacokinetic profile to the high DAR ADC, thereby reduces non-specific clearance and improves in vivo potency.

## 6. Absorption, Distribution, Metabolism, and Excretion (ADME) of ADCs

As discussed in the sections above, considerable advancements in next generation ADCs are anticipated to further explore both the chemical and biological design elements for ADCs. This includes, but is not limited to, antibody engineering to facilitate direct site-specific conjugation, modification of conjugation chemistry and introduction of novel linker compositions to confer enhanced stability, as well as, the exploration of additional existing and novel chemical entities/modalities as conjugates to increase the pharmacological applications of ADCs. Many of these advancements to design better molecules are intricately interdependent with optimizing or improving the ADME drug-ability properties of ADCs, such as linker-payload stability, distribution, and pharmacokinetics (PK). This is because defining the exposure-response relationship for both safety and efficacy has been intimately tied with ADC peripheral PK, target tissue or site of action concentration and the disposition of the payload at the intended site. The criticality of understanding the exposure-response relationship and therapeutic index (TI) for ADCs is exemplified by Mylotarg^®^ (gemtuzumab ozogamicin) which is composed of a CD33 mAb linked to the cytotoxic drug calicheamicin via an acid-liable hydrazine linker. While initially approved for the treatment of acute myeloid leukemia (AML) in 2000, it was pulled from the market at the request of regulatory agencies in 2010 due to safety concerns and the failure to reproduce the clinical benefit connected to linker stability in AML patients. Following additional interrogation of exposure-response relationships, examining alternative lower dosing and scheduling, Mylotarg found a path back to the market and received a new FDA approval for newly diagnosed CD-33 positive acute AML patients in 2017.

Dissecting the ADME properties of ADCs is a complex endeavor given the unique properties of each component within the molecules. ADC ADME involves delineating the intertwined properties of linker-payload stability, pharmacokinetics, clearance, metabolism, and disposition of mAb, conjugate (i.e., small molecule chemical entity for traditional ADCs), as well as, the ADC entity itself. The ADME properties of ADCs are influenced by the mAb, target antigen, linker, site of conjugation, DAR number, and the conjugated species (i.e., payload). Table 2 summarizes the various types of in vitro and in vivo studies to characterize ADC ADME.

The antibody component of ADCs is the primary driver of the slow clearance, long systemic half-life and restricted tissue distribution of these modalities compared to their payload counterparts. Similar to mAbs, the properties related to target (expression pattern, density and turnover), as well as, antibody structure (including physiochemical properties, FcRn binding, Fcγ receptor interactions and isotype) that affect antibody PK and disposition also impact ADCs. An anti-drug antibody (ADA) or neutralizing antibody (Nab) response against the therapeutic antibody component can affect the PK profile and shorten the half-life of the ADCs in the body [9,19]. Nevertheless, idiotype networks have an important biological role in avoiding the expansion of autoreactive B or T-cells [137,138]. Uniquely, ADC PK is also impacted by linker composition, chemical nature of the payload and DAR. These are both speculated to affect the physiochemical properties of the ADC which are linked to the PK and clearance of the molecules. For example, ADCs with high DAR values have been shown to aggregate and have higher clearance rates than their unconjugated mAb counterparts or lower DAR species [59,129,139]. Similarly, decreasing the hydrophobicity and improving the hydrophilicity of the linker component within an anti-CD70 and anti-HER2 mAbs improved the exposure and changed the disposition of the ADCs [129,140]. 

In addition to the antibody, the linker and payload components of ADCs are also subject to their own clearance mechanisms. The stability of the linker to premature release of the payload in the systemic circulation has been demonstrated to be a critical ADC ADME component for determining the exposure-response and exposure-toxicity relationships [141]. From a stability perspective, well-behaved ADCs should only release the payload in the intended target tissue to minimize payload toxicity to unintended tissue and maximize efficacy in target tissues/organs, especially with payloads with cytotoxic properties. The ADC linker stability is noted to be a challenge due to the long circulating half-life (days to weeks) imparted by the mAb component resulting in the continuous assault of the linker to endogenous proteases. Mechanistically, linker stability can be evaluated both in vitro using plasma/serum incubations and in vivo following administration to multiple species by following the formation of the released payload and DAR changes over time. As covered above, linker composition continues to be an intense area of focus in the development of ADCs. In terms of the payload, initial reports with limited chemical entities suggested that type of payloads did not impact the PK of ADCs; however, more recent studies of conjugation sites and of site-specific conjugations have demonstrated the connectivity of the site of conjugation with various payloads to impact ADC PK and disposition [142,143]. Engineering ADCs for site-specific conjugation to control the DAR and PK has shown some evidence of improving the TI in non-clinical oncology studies [144]. Approaches such as engineered cysteines, unnatural amino acids, and the inclusion of tags (i.e., selenocysteine, aldehyde, or glutamine) continue to be intense areas of research for the application of site-specific payload conjugation to optimize ADC ADME.

Like mAbs, ADCs are likely trafficked via the vascular and lymphatic systems. The biodistribution of ADCs follows that of the antibody component. ADC are removed from the systemic circulation by target-mediated drug disposition (TMDD); thus, highly vascularized organs or tissues that express the target antigen are involved in the clearance of ADCs from the periphery. The TMDD is believed to be followed by intracellular trafficking of the target: ADC complex to lysosomes where degradation of the ADC occurs, and the payload is released from the mAb to elicit its activity. In addition, nonspecific uptake of ADCs by pinocytosis also facilitates their systemic depletion. This form of uptake could lead to degradation and/or recycling of the ADC by the neonatal Fc receptor (FcRn). Indeed, in terms of tissue distribution the preponderance of ADCs are observed in four organs including the liver, kidneys, lungs, and skin [145,146]; however, the amount in each tissue differs between ADCs based on their target binding and physiochemical properties [147]. Importantly, irrespective of the mode of ADC degradation, the payload or chemical moiety can be released into the blood. The unconjugated payload is expected to follow the biodistribution pattern of a typical small molecule drug which is widely distributed throughout the tissues.

The elimination of ADCs involves two processes. First, intracellular catabolism through proteolysis in the tissues (i.e., TMDD- or pinocytosis-mediated). Second, complete deconjugation of the payload which can result in both mAb or mAb with a partial linker along with free drug [148]. While the mAb based species are expected to follow catabolism through the same mechanisms as the ADC, there is increased attention in the elimination of the unconjugated payload under conditions of impairment of renal and hepatic processes. For example, a study of brentuximab vedotin showed that the major route of MMAE excretion was through the feces (~72%) and the remaining MMAE was recovered in urine in humans [149]. Given these data, the relationship of hepatic and renal insufficiency to ADC exposure is an important aspect of clinical development.

Another area of intense research is dissecting the noted phenomenon of resistance against ADCs. A few mechanisms of resistance have been noted including to the antibody portion of the ADCs by mutation and/or down-regulation of the target antigen, as well as, to the payload via drug efflux transporters that remove the payload from cells [150]. Changes in the intracellular processing of ADCs through alterations of the linker cleavage caused by lysosomal or endosomal abnormalities can also significantly affect the PK profiles. These changes impair the release of payloads in the cytosol and consequently affect the therapeutic indexes of the ADCs [150].

## 7. Conjugate Developability, Formulations, and Characteristics

An antibody conjugate combines an inherently complex antibody with a small synthetic molecule drug to create an even more complex large molecule. Despite the relatively small addition in molecular weight, the small molecule drug has a profound impact on the characteristics and properties of the conjugate.

Over the last decade, significant advancements in analytical methods have been made to characterize ADCs and have been extensively reviewed [151,152,153,154]. These methods have focused on the major ADC attributes such as DAR, drug load distribution, residual linker-payload and related impurity levels, in addition to typical attributes for antibodies such as aggregation level, charge variants, and host cell protein level. The DAR number has a strong influence on the properties of ADCs. Currently, many of the ADCs in the clinic have DAR numbers in the range of 2–4, although ADCs with higher DAR numbers have also been reported [31,155,156,157,158]. For stochastically conjugated ADCs, the small molecule drug is covalently linked to either the lysine or the interchain cysteine residues of the antibody, resulting in a heterogeneous mixture with various DAR species which are more difficult to characterize and control. For example, as many as 40 lysines were found to be partially modified in a lysine conjugated ADC molecule using LC-MS and peptide mapping methods [159]. The aforementioned site-specific conjugation approaches have drastically improved the DAR homogeneity albeit process development is required to further control the remaining heterogeneity during the production process [160]. At present, nearly all the antibody conjugate characterization literature has focused on antibody-small organic molecule conjugates. Based on the molecular nature of each payload, chromatographic and electrophoretic methods as well as spectroscopic methods have been commonly employed in DAR determination along with mass spectrometer method which provides more detail. The analytical methods will continue to evolve as the payload expands to nucleic acids which possess very different properties compared with small organic molecules [120,161].

It is well-documented that the addition of a small molecule drug to an otherwise soluble and stable antibody can cause aggregation and other physicochemical instability in the ADC [162,163]. This is not only because many of the small molecule drugs are bulky and hydrophobic in nature leading to a significant increase in the hydrophobicity of the ADC, but also because the conjugation can induce perturbations to secondary and tertiary structures of the antibody resulting in reduced conformational stabilities. To this point, a systematic study of trastuzumab, trastuzumab-MCC conjugate intermediate, and trastuzumab-DM1 found that both conjugates suffered decreased thermal stability and increased aggregation compared with trastuzumab [164]. Recently, the impact of drug conjugation on intra- and intermolecular interactions of trastuzumab-DM1 compared with trastuzumab was studied and the results confirmed that the lower colloidal stability and higher aggregation propensity for trastuzumab-DM1 are attributed to both reduced repulsive charge interaction and increased hydrophobicity [165]. Multiple publications have reported a more pronounced conjugation destabilizing effect on interchain cysteine conjugated ADCs and an inverse correlation between the drug load and stability [166,167,168,169,170]. Consistent with the findings on the interchain cysteine conjugated ADCs, the high DAR species in trastuzumab-DM1, a lysine conjugate, have also been found to be less stable and more prone to aggregation than the low DAR species [171]. Site-specific conjugation approaches with carefully chosen conjugation sites are expected to have less negative impact on stability and aggregation propensity of ADCs [64,65,172]. Most site-specific conjugates in the clinical pipeline have homogeneous DAR of 2 resulting in reduced hydrophobicity and aggregation compared to stochastic conjugates of average DAR of 3.5–4 where DAR species range from 0 to 8 or higher. In order to achieve sufficient efficacy with a relatively low DAR number, potent payloads such as PBD, a MDR1-resistant maytansine payload, and an auristatin payload Aur0101 have been developed. However, the DAR 2 site-specific conjugates with PBD and Aur0101 have shown limited therapeutic index in clinic thus far [173,174,175,176], while the clinical data for the DAR 2 maytansine ADC is pending [177]. While extensive characterization studies have been reported for antibody conjugated to cytotoxic payloads, there is a scarcity of literature on the molecular properties of conjugates with non-toxic small organic molecule payloads and nucleic acids. The optimal DAR number and solution property of conjugates with oligonucleotides, which are highly charged and significantly larger than small organic molecules, remains to be determined.

In addition to all the issues encountered during antibody formulation development, the formulation development of ADC drugs must find suitable pH and excipient conditions to simultaneously maintain the stability of the antibody, the linker, and the small molecule drug (reviewed in [162,163]). Even if there is an in-depth understanding of the stability of the parental antibody in aqueous solution, its physical stability may change upon conjugation in the presence of organic solvent or through possible cross-linking mechanism, and its chemical stability may depend on the conjugation method [178]. An example is the light-sensitivity in a model ADC using trastuzumab whereas trastuzumab itself does not show such sensitivity [179]. While much is known in the literature regarding the chemical stability of monoclonal antibodies [180] and the data on commonly used cytotoxic linker-payloads is accumulating [153,181,182], novel and non-toxic linker-payloads including siRNA will require a clear understanding of their degradation pathways in order to form control strategies during drug development process, similar to what has been demonstrated on payload metabolism [183]. Therefore, a comprehensive evaluation of the combined system will always be necessary for novel ADCs.

All the current ADC drugs on the market are lyophilized products suitable for intravenous administration. Such a freeze-dried state protects the ADCs from chemical degradation and aggregation which occur under long-term solution storage conditions. Although interest has been growing for liquid formulation based on the increased experience with ADCs and the improved solubility and stability of the new generation of ADCs, few such feasibility studies have been reported in the literature to date. It can be particularly challenging to prevent payloads from falling off the antibody over a long period of time in solution. In addition, the currently approved ADC drugs are reconstituted to 0.25–20 mg/mL in solution, significantly below the concentrations for most therapeutic antibody products. While the above is a viable approach for intravenous administration commonly used for oncology therapies, the emerging non-oncology application of the ADCs will likely demand stable liquid formulation and subcutaneous administration as commonly expected for many antibody drugs to increase convenience for patients. This growing trend exerts pressure on linker-payload design and conjugation methods in addition to the properties of the parental antibodies, as well as on formulation and device development. The current pre-clinical data for non-oncology ADCs suggest that the ADC doses might not be significantly lower than those for antibodies [41,45,47,52]. The clinical efficacy and therapeutic index of the non-oncology ADCs will ultimately determine the dose requirement and the appropriate drug product concentration.

## 8. Conclusions

Antibody conjugates in oncology have thus far delivered several successfully approved therapeutics. Extensive research into novel payloads, more developable linkers and conjugation chemistries further enable the field of oncology conjugates to cross the finish line. These learnings and advances also help propel the next generation of conjugates for non-oncology indications. Additional challenges such as in vivo stability, formulation and delivery will drive the field to seek solutions to broaden the therapeutic horizon to include payloads like nucleic acids. Overall, the rise of non-oncology ADC therapeutics offers a huge opportunity for innovation at multiple fronts of drug discovery and development for years to come.

## Figures and Tables

**Table 1:** FDA approved antibody-drug-conjugates (ADCs).

ADC Product	Indications	Approval Date	Target Antigen	Antibody Conjugation	Average Drug Antibody Ratio (DAR)	Linker	Payload
Mylotarg^®^ (Gemtuzumab ozogamicin)	Relapsed AML	2001, withdrawn 2010; (reapproved 2017)	CD33	Humanized IgG4—lysine	2–3	Hydrazone	Calicheamicin
Adcetris^®^ (Brentuximab vedotin)	Relapsed HL and sALCL	2011	CD30	Chimeric IgG1—cysteine	~4	Dipeptide cleavable (Val-Cit)	Monomethyl auristatin E (MMAE)
Kadcyla^®^ (Trastuzumab emtansine)	HER2 + metastatic breast cancer	2013	HER2	Humanized IgG1—lysine	3.5	Thioether Non-cleavable	Emtansine (DM1)
Besponsa^®^ (Inotuzumab ozogamicin)	Relapsed or refractory CD22 + B-ALL	2017	CD22	Humanized IgG4—lysine	~4	Hydrazone	Calicheamicin
Polivy^®^ (Polatuzumab vedotin)	Relapsed or refractory DLBCL	2019	CD79b	Humanized IgG1—cysteine	3.5	Dipeptide cleavable (Val-Cit)	Monomethyl auristatin E (MMAE)
Enhertu^®^ (fam-trastuzumab deruxtecan-nxki)	HER2 + unresectable metastic breast cancer	2019	HER2	Humanized IgG1—cysteine	7–8	Tetrapeptide cleavable (Gly-gly-Phe-Gly)	Exatecan derivative (Dxd)

**Table 2:** ADME Characterization approaches for ADCs and their constituents.

Species	ADME Information
Antibody	Determine PK-dose relationship in vivo Characterize target affinity/specificity in vitro, target expression/turnover in vivo, unintended or off-target binding in vitro and in vivo
ADC	Linker Component (1) Characterize linker stability and kinetics of catabolism in vitro and in vivo across species (2) Evaluate nature of the released species (active payload and its catabolites) Conjugation Site (1) Evaluate influence of conjugation site on linker stability in vitro and in vivo (2) Determine the effect of conjugation site on PK DAR (1) Determine in vivo PK and disposition with heterogenous and homogenous DAR species
Payload	Metabolite identification, characterize DDI potential (CYP inhibition, induction and reaction phenotypes) P-gp substrate or inhibitor Characterize non-P-gp transporters Plasma protein binding
